# Ubiquitination of transcription factors in cancer: unveiling therapeutic potential

**DOI:** 10.1002/1878-0261.70033

**Published:** 2025-04-14

**Authors:** Dongha Kim, Hye Jin Nam, Sung Hee Baek

**Affiliations:** ^1^ Department of Anatomy, College of Medicine The Catholic University of Korea Seoul Korea; ^2^ Therapeutics and Biotechnology Division Korea Research Institute of Chemical Technology Daejeon Korea; ^3^ Department of Medicinal Chemistry and Pharmacology University of Science and Technology Daejeon Korea; ^4^ Creative Research Initiatives Center for Epigenetic Code and Diseases, School of Biological Sciences Seoul National University Korea

**Keywords:** cancer, deubiquitinase, E3 ligase, PROTAC, transcription factor, ubiquitination

## Abstract

Transcription factors, pivotal in gene expression regulation, are essential in cancer progression. Their function is meticulously regulated by post‐translational modifications, including ubiquitination. This process, which marks proteins for degradation, can either enhance or inhibit the function of transcription factors, contingent on the context. In cancers, dysregulated ubiquitination of transcription factors contributes to the hallmark of uncontrolled growth and survival of tumors. For example, tumor suppressors such as p53 might be degraded prematurely due to abnormal ubiquitination, causing genomic instability. On the other hand, oncogenic transcription factors may gain stability via ubiquitination, thus facilitating tumorigenesis. Targeting the ubiquitin–proteasome system (UPS) therefore could be a viable therapeutic approach in cancer. Emerging treatments aim to block the ubiquitination of oncogenic transcription factors or to stabilize tumor suppressors. This review underscores the critical impact of transcription factor‐altered ubiquitination on cancer progression. Additionally, it outlines innovative therapeutic approaches that involve inhibitors or drugs directed at specific ubiquitin E3 ligases and deubiquitinases (DUBs) that regulate transcription factor activity.

## Graphical abstract

In cancer, dysregulated ubiquitination of transcription factors contributes to the uncontrolled growth and survival characteristics of tumors. Tumor suppressors are degraded by aberrant ubiquitination, or oncogenic transcription factors gain stability through ubiquitination, thereby promoting tumorigenesis. Therefore, we highlight innovative therapeutic approaches that involve inhibitors or drugs directed at specific ubiquitin E3 ligases and deubiquitinases (DUBs) that regulate transcription factor activity.

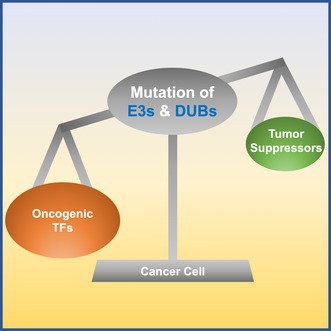

**Abbreviations**
AKT: Ak strain transforming
AR: androgen receptor
ATM: ataxia telangiectasia mutated protein
ATR: ataxia telangiectasia and Rad3‐related protein
ccRCC: clear cell renal cell carcinoma
CDKs: cyclin‐dependent kinases
CDT1: chromatin licensing and DNA replication factor 1
CHIP: carboxy terminus of HSP70‐interacting protein
CHK: checkpoint kinase
CK1: creatine kinase1
CRBN: cereblon
CRLs: cullin‐RING E3 ubiquitin ligases
CSCs: cancer stem cells
CUL4A: cullin‐4A
CYLD: cylindromatosis
DNA‐PK: DNA‐dependent protein kinase
DUBs: deubiquitinases
Dvl: Disheveled
E4F1: E4F Transcription Factor 1
ER: endoplasmic reticulum
ERK: extracellular signal‐Regulated kinase
ESCs: embryonic stem cells
FBW: F‐box and WD repeat domain‐containing
FBXW: F‐box and WD repeat domain‐containing
FDA: food and drug administration
GSK‐3β: glycogen synthase kinase‐3β
HAUSP: Herpes‐specific ubiquitin specific protease
HECT: homologous to the E6‐AP carboxyl terminus
HIF‐1α: hypoxia inducible factor 1α
HREs: hypoxia‐response elements
IKK: IκB kinase
iPSC: induced pluripotent stem cell
ITCH: itchy E3 ubiquitin protein ligase
IκB: inhibitory κB
JADE1: Jade Family PHD Finger 1
KLHL12: kelch‐like protein 12
MCL: mantle cell lymphoma
MEFs: mouse embryonic fibroblasts
NARF: nuclear prelamin A recognition factor
NEDL: neuronal HECT‐type ubiquitin‐protein isopeptide ligase
NHL: non‐Hodgkin lymphoma
NPC: neural progenitor cell
OCT4: octamer‐binding transcription factor 4
OTUD: OTU deubiquitinase
OTULIN: OTU deubiquitinase with linear linkage specificity
PARP: poly(ADP‐ribose) polymerase
PD‐L1: programmed cell death ligand 1
PHD: prolyl hydroxylase domain
PJA2: Praja ring finger ubiquitin ligase 2
PLK3: polo‐like kinase 3
POI: protein of interest
POSTN: periostin
PP2A: protein phosphatase 2A
PROTACs: PROteolysis TArgeting Chimeras
PTMs: post‐translational modifications
RANBP2: RAN binding protein 2
RNF: ring finger protein
SCF: SKP1‐cullin 1‐F‐box
SET7/9: SET domain containing lysine methyltransferase 7/9
SIAH: seven in absentia homolog
SKP2: S‐phase kinase associated protein 2
SMURF1: SMAD specific E3 ubiquitin protein ligase 1
SOX2: SRY‐Box transcription factor 2
SPOP: speckle‐type POZ protein
STAT3: signal transducer and activator of transcription 3
TCF/LEF: T‐cell factor/lymphoid enhancer factor
TFs: transcription factors
TPD: targeted protein degradation
TRIM: tripartite motif
UBE2D1: ubiquitin‐conjugating enzyme E2 D1
UBE2K: ubiquitin‐conjugating enzyme E2K
UCHL5: USP14 and ubiquitin C‐terminal hydrolase L5
UCHs: ubiquitin C‐terminal hydrolases
UPS: ubiquitin‐proteasome system
USPs: ubiquitin‐specific proteases
VHL: Von Hippel–Lindau
WWP2: WW domain containing E3 ubiquitin protein ligase 2
β‐TrCP: β‐transducin repeats‐containing proteins

## 1 Introduction

The ubiquitin–proteasome system (UPS) is a cellular machinery responsible for the degradation of proteins. Ubiquitination, a pivotal post‐translational modification, is a multistep process that involves the covalent attachment of a small, highly conserved protein called ubiquitin to a target protein [1, 2, 3]. The UPS includes the 26S proteasome, a large, multiprotein complex, and a series of enzymes involved in ubiquitin conjugation. The 26S proteasome is a barrel‐shaped structure that unfolds and degrades proteins into small peptides, which can then be recycled or presented to the immune system [4, 5].

Ubiquitin conjugation is a complex enzymatic cascade that involves three key enzymes: ubiquitin‐activating enzyme (E1), ubiquitin‐conjugating enzyme (E2), and ubiquitin ligase (E3). The specificity of ubiquitination is largely determined by the E3s. Different E3s recognize specific substrates and catalyze the formation of distinct ubiquitin chain patterns, thereby controlling the fate of the target protein [6]. Deubiquitination is a cellular process that serves as a counterbalance to ubiquitination [7]. While ubiquitination marks proteins for degradation or other cellular functions, deubiquitination removes these marks, effectively reversing the process.

Maintaining protein homeostasis through the UPS is essential for normal cellular function. When this delicate balance is disrupted, misfolded or aggregated proteins accumulate, which may be implicated in a variety of human diseases, including cancer [8, 9]. The complex relationship between protein quality control and cancer development has become the focus of biomedical research [10, 11]. Understanding how changes in protein homeostasis contribute to tumorigenesis will allow us to identify new therapeutic targets and develop more effective cancer treatments. In this review, we highlight the importance of the UPS in cancer development and progression, focusing particularly on the ubiquitination or deubiquitination processes of transcription factors involved in cancer.

## 2 Ubiquitination of transcription factors in cancer

**Fig. 1:**
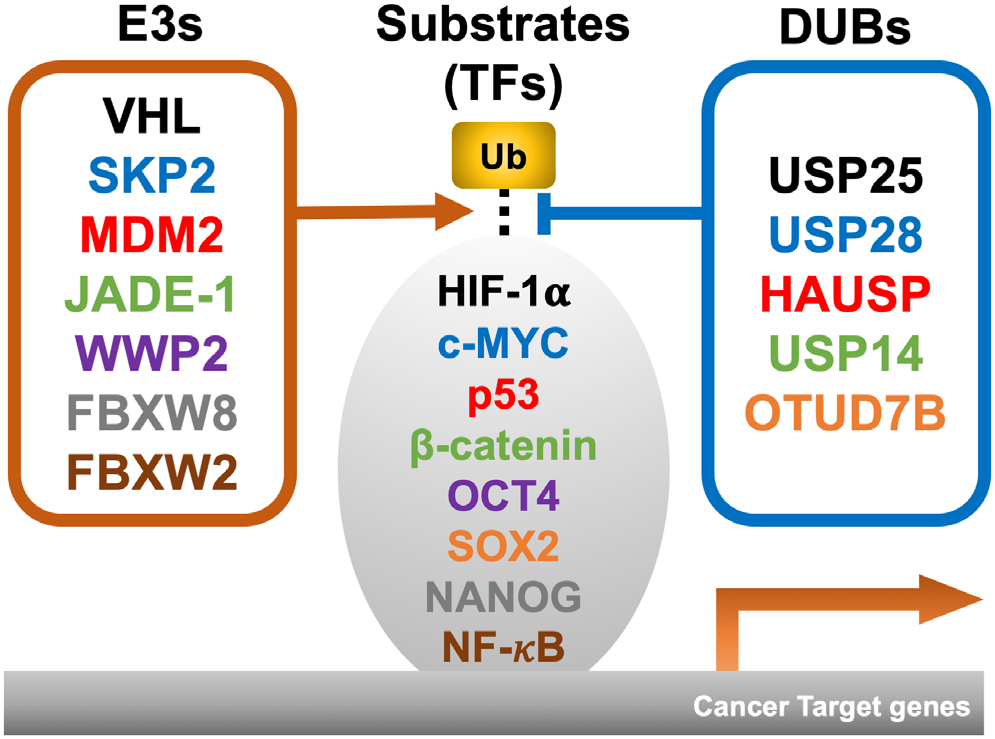
Transcriptional regulation of cancer target genes through ubiquitination of transcription factors. The stability of critical transcription factors, which regulate cancer target genes in various cancer types, is specifically controlled by certain E3s and DUBs. Colors represent E3s and DUB pairs that target each transcription factor.

The research findings from the examples above highlight the critical role of ubiquitination in cancer development and metastasis by targeting various substrate proteins and regulating their stability. Among these, the ubiquitination of transcription factors (TFs), which directly control the expression of cancer‐related genes, is particularly vital in cancer progression (Fig. 1). Therefore, it is crucial to understand ubiquitination in TFs that are key drivers of cancer development.

### 2.1 HIF‐1α

Under conditions of rapid proliferation, tumor cells encounter a state of oxygen deprivation, termed hypoxia. To compensate for the increased energy demands associated with this proliferative state, tumor cells exhibit metabolic reprogramming. This reprogramming process is principally orchestrated by the HIF‐1α transcription factor. Under normoxic conditions, HIF‐1α protein undergoes degradation, but it becomes stabilized in hypoxic environments. Once stabilized, HIF‐1α translocates to the nucleus, where it regulates a broad array of genes that affect various cellular processes, thereby promoting tumor survival, proliferation, and metastasis. Furthermore, HIF‐1α orchestrates metabolic reprogramming by favoring glycolysis for ATP production in cancer cells [12].

HIF‐1α stability is mainly regulated by ubiquitination. Under normoxic conditions, HIF‐1α undergoes rapid proteasomal degradation mediated by the VHL. This degradation process involves the hydroxylation of HIF‐1α by prolyl hydroxylase domains (PHDs) at specific proline residues. Hydroxylated HIF‐1α interacts with VHL, targeting HIF‐1α for ubiquitination and subsequent proteasomal degradation [13]. In hypoxic conditions, PHD activity is inhibited by decreased oxygen levels. Consequently, HIF‐1α remains unhydroxylated and unable to bind VHL. This prevents HIF‐1α degradation, allowing it to accumulate and translocate to the nucleus [14]. In the nucleus, HIF‐1α dimerizes with its partner protein, HIF‐1β, forming the active HIF‐1 transcription complex. This complex binds to hypoxia‐response elements (HREs) in the promoter regions of target genes, initiating their transcription [15]. The intricate regulation of HIF‐1α stability ensures that its activity is tightly controlled in response to changes in oxygen levels, allowing cells to adapt to hypoxic conditions and maintain cellular homeostasis. Dysregulation of HIF‐1α stability, particularly its stabilization under normoxic conditions, is a hallmark of cancer and contributes to tumor progression, angiogenesis, and metastasis [16]. Understanding the mechanisms governing HIF‐1α stability is crucial for developing novel therapeutic strategies targeting HIF‐1α signaling pathways in cancer and other diseases.

VHL is considered a tumor‐suppressor protein [17]. Dysregulation of VHL, often resulting from gene mutations or deletions, is frequently observed in various cancers, including clear cell renal cell carcinoma (ccRCC), hemangioblastomas, and pheochromocytomas.

While functional inactivation of the VHL and the consequent constitutive activation of the HIF pathway are well‐established as oncogenic drivers in ccRCC [18, 19], the precise mechanisms underlying their contribution to metastatic progression remain elusive. Recent studies have shed light on how VHL mutations can promote metastatic dissemination. These findings reveal that VHL mutations drive metastasis through the production of soluble metastatic mediators, such as periostin (POSTN), an HIF‐1α target gene [20]. POSTN, in turn, enhances the motility of VHL wildtype cells and facilitates tumor cell dissemination by disrupting the vasculature. This highlights the role of VHL mutations in driving metastasis through intercellular communication within the intratumoral heterogeneous environment of ccRCC.

The regulation of HIF‐1α stability extends beyond ubiquitination mediated by the PHD–VHL axis, encompassing a diverse array of post‐translational modifications (PTMs). Among these PTMs, phosphorylation stands out as a prominent regulatory mechanism. A multitude of kinases, including glycogen synthase kinase‐3β (GSK‐3β), polo‐like kinase 3 (PLK3), cyclin‐dependent kinases (CDKs), and ataxia telangiectasia mutated protein (ATM), phosphorylate HIF‐1α, exerting a profound influence on its stability [21]. GSK‐3β‐mediated phosphorylation of HIF‐1α facilitates its targeting by the FBW7 complex, leading to proteasomal degradation [22, 23]. GSK‐3β is an important regulator that complements the PHD–VHL system in controlling HIF‐1α stability. PLK3‐mediated phosphorylation of HIF‐1α results in its destabilization, akin to the regulatory mechanism of GSK‐3β [24]. Conversely, phosphorylation can enhance the stability of the HIF‐1α protein. CDK1‐associated phosphorylation of HIF‐1α prevents its lysosomal degradation [25]. The levels of HIF‐1α are modulated by CDK activity across different cell‐cycle phases, facilitating cell proliferation while concurrently triggering the adaptive HIF‐1α transcriptional response. Additionally, ATM phosphorylates HIF‐1α in response to hypoxia, thereby enhancing both its stability and transcriptional activity.

HIF‐1α methylation is also implicated in its stability and the progression of human cancer. The SET domain containing lysine methyltransferase 7/9 (SET7/9)‐mediated methylation of HIF‐1α at the K32 site contributes to its instability in the nucleus under normoxic conditions [26]. Conversely, under hypoxia, the mRNA and protein expression of the lysine‐specific demethylase 1 (LSD1) demethylase increases, leading to the demethylation of HIF‐1α, which in turn enhances its stability. Notably, methylation‐defective HIF‐1α knock‐in mice exhibit increased tumor growth and angiogenesis. Mutations that impair HIF‐1α methylation, such as serine 28 tyrosine (S28Y) and arginine 30 glutamine (R30Q) within the SET7/9 targeting consensus sites, have been identified in human cancers, suggesting the critical role of methylation in cancer biology.

### 2.2 c‐MYC

c‐MYC, a pivotal transcriptional factor, plays a critical role in regulating cell growth, differentiation, and tumorigenesis. Its protein levels are tightly controlled by the UPS and are frequently dysregulated in various cancers [27]. As a highly unstable protein, c‐MYC is rapidly degraded in cells. Impaired c‐MYC protein degradation, leading to its accumulation, is implicated in the uncontrolled proliferation observed in numerous cancers [28]. Several E3 ubiquitin ligases and deubiquitinating enzymes, including FBW7, β‐transducin repeats‐containing proteins (β‐TrCP), S‐phase kinase associated protein 2 (SKP2), USP28, and USP36, have been identified as key regulators of c‐MYC stability.

FBW7, a key component of the SKP1‐cullin 1‐F‐box (SCF)‐type E3 ubiquitin ligase complex, regulates the stability of c‐MYC. c‐MYC stability is contingent upon its phosphorylation at specific residues, threonine 58 (T58) and serine 62 (S62) [29]. Following growth factor stimulation, c‐MYC is stabilized through phosphorylation at the S62 site by ERK and/or CDKs. This S62 phosphorylation serves as a priming event for subsequent phosphorylation at T58 by GSK‐3β. Pin1‐mediated isomerization of T58‐phosphorylated c‐MYC facilitates its dephosphorylation at S62 by protein phosphatase 2A (PP2A)‐B56α. The dephosphorylated c‐MYC protein is then recognized by the SCF^FBW7^ E3 ubiquitin ligase complex and targeted for degradation by the 26S proteasome [30]. Interestingly, mice expressing a T58A mutant of c‐MYC in their mammary glands exhibited mammary carcinoma, elevated genomic instability, and suppressed apoptosis [29]. These findings suggest that the phosphorylation status of T58 significantly influences the function of c‐MYC and its ability to drive tumorigenesis in the mammary gland. In certain contexts, c‐MYC stability can be enhanced by ubiquitination. The SCF^β‐TrCP^ complex is implicated in promoting c‐MYC stability on recovery from S phase arrest [31]. Both FBW7 and β‐TrCP mediate direct ubiquitination of the N‐terminal region of c‐MYC; however, SCF^β‐TrCP^ generates heterogeneous poly‐ubiquitin chains composed of K63 and K48 linkages, while SCF^FBW7^ exclusively forms K48‐linked chains.

SKP2 facilitates the poly‐ubiquitination and subsequent degradation of c‐MYC [32]. Unlike FBW7, SKP2‐mediated regulation of c‐MYC degradation does not appear to be dependent on c‐MYC phosphorylation. The role of SKP2 in c‐MYC regulation is complex. While SKP2 promotes c‐MYC degradation, it also functions as a transcriptional coactivator of c‐MYC, enhancing its transcriptional activity.

The deubiquitinating enzyme USP28 has been identified as a regulator of c‐MYC stability [33]. USP28 interacts with c‐MYC through FBW7α and stabilizes c‐MYC in human tumor cells. Elevated expression levels of USP28 are observed in colon and breast carcinomas, and the stabilization of c‐MYC by USP28 is critical for tumor cell proliferation. USP36, another deubiquitinating enzyme, has been implicated in regulating c‐MYC stability [34]. Elevated expression levels of USP36 are observed in specific subsets of human breast and lung cancers. While USP36 preferentially interacts with the nucleolar FBW7γ variant, it can also counteract c‐MYC degradation mediated by both FBW7γ and the nucleoplasmic FBW7α.

### 2.3 p53

The tumor suppressor p53 is a critical transcriptional factor that regulates cell cycle arrest and apoptosis. Post‐translational modifications, including acetylation, methylation, phosphorylation, neddylation, sumoylation, and ubiquitination, significantly influence the stability of p53 and transcriptional activity. Ubiquitination of p53, mediated by the UPS, markedly impacts its protein levels and turnover. Both mono‐ubiquitination and poly‐ubiquitination play distinct and essential roles in the functions of p53.

MDM2 is the primary E3 ubiquitin ligase responsible for p53 ubiquitination and subsequent degradation. p53 and MDM2 engage in a negative feedback loop, with p53 promoting the expression of MDM2 during normal cellular conditions to maintain low p53 levels [35]. Phosphorylation of p53 at serine 15 and serine 20 in response to DNA damage and other cellular stressors by ATM, ataxia telangiectasia and Rad3‐related protein (ATR), DNA‐dependent protein kinase (DNA‐PK), Checkpoint kinase (CHK)1, and CHK2 is believed to disrupt the MDM2‐p53 interactions, thereby stabilizing p53 [36].

MDM2 is capable of catalyzing both monoubiquitination and polyubiquitination of p53. As monoubiquitination of p53 induces its nuclear export, regulating MDM2 levels presents an additional mechanism for controlling p53 activity [37]. During unstressed conditions, low levels of MDM2 may promote p53 monoubiquitination, leading to its nuclear export and removal from transcriptional targets.

The transcription factor E4F Transcription Factor 1 (E4F1) is capable of ubiquitinating p53, although this modification does not affect p53 stability [38]. E4F1 is an atypical ubiquitin ligase, as it lacks the commonly associated RING or HECT domain. Instead, its enzymatic activity resembles that of the SUMO E3 ligase RAN binding protein 2 (RANBP2) [38]. E4F1‐mediated ubiquitination of p53 enhances its association with chromatin, specifically coinciding with p53‐mediated transcriptional activation of genes involved in cell cycle arrest, but not apoptosis.

Herpes‐specific ubiquitin‐specific protease (HAUSP) is a deubiquitinating enzyme that directly stabilizes p53 by removing ubiquitin modifications [39]. However, the effects of HAUSP on the p53‐MDM2 pathway are multifaceted. Given that HAUSP also deubiquitinates MDM2, its stability in normal cells is dependent on HAUSP activity [40]. Surprisingly, nearly complete ablation of HAUSP can paradoxically lead to the stabilization and activation of p53.

### 2.4 β‐Catenin

The Wnt signaling pathway plays a pivotal role in a wide range of cellular processes, including embryonic development, cell proliferation, migration, stem cell regulation, and pigment biosynthesis [41]. In the Wnt pathway, ubiquitination serves as a primary regulatory mechanism for controlling β‐catenin expression levels. In the absence of Wnt ligands, cytosolic β‐catenin is phosphorylated by creatine kinase1 (CK1) at S45 and GSK3 at S33, 37, and T41, leading to its ubiquitination by the E3 ligase β‐TrCP and subsequent degradation by the 26S proteasome [42].

Upon stimulation of Frizzled receptors and activation of Disheveled (Dvl), β‐catenin is stabilized by escaping ubiquitination [43]. The accumulated β‐catenin then translocates to the nucleus, where it initiates the expression of Wnt target genes with T‐cell factor/lymphoid enhancer factor (TCF/LEF) transcription factors [44]. Dysfunctional Wnt signaling has been implicated in a variety of human cancers, and elevated β‐catenin levels are associated with tumor growth [44]. Constitutive activation of the Wnt/β‐catenin signaling pathway, often driven by mutations in Wnt signaling genes or aberrant activation of Wnt receptors, has been implicated in tumorigenesis in various tissues, including the colon, liver, skin, breast, and bone marrow [45].

Structure that is a member of the seven in absentia homolog (SIAH)1, another E3 ubiquitin ligase targeting β‐catenin, mediates ubiquitination at specific lysine residues, K666 and K671, in conjunction with the E2 enzyme ubiquitin‐conjugating enzyme E2 D1 (UBE2D1). Elevated SIAH1 expression results in cytoplasmic β‐catenin ubiquitination, regardless of β‐catenin phosphorylation [46]. Additionally, nuclear β‐catenin can be ubiquitinated by Jade Family PHD Finger 1 (JADE1), leading to its degradation [47]. JADE1 also targets both phosphorylated and nonphosphorylated β‐catenin, thereby regulating canonical Wnt signaling in both the Wnt‐off and Wnt‐on states.

In addition to β‐catenin, other key components of the Wnt signaling pathway, such as Dvl and TCF/LEF, are also subject to ubiquitination by various E3 ubiquitin ligases. Dvl is a pivotal component of the canonical Wnt signaling pathway as a scaffold protein that facilitates interactions between receptors and downstream signaling components. Several E3 ubiquitin ligases, including kelch‐like protein 12 (KLHL12)‐Cullin‐3, pVHL, neuronal HECT‐type ubiquitin‐protein isopeptide ligase (NEDL)1, Malin, and itchy E3 ubiquitin protein ligase (ITCH), have been identified as regulators of Dvl stability [43, 48, 49, 50]. These E3 ligases promote the ubiquitination and degradation of Dvl under various physiological conditions.

K63‐linked polyubiquitination of Dvl has been reported, and depletion of CYLD leads to a significant increase in K63‐linked polyubiquitination of Dvl, consequently enhancing Wnt‐induced accumulation of β‐catenin and activation of Wnt target genes [51]. CYLD negatively regulates Wnt/β‐catenin signaling by disassembling K63‐linked polyubiquitin chains on Dvl, providing an additional mechanism for controlling Wnt signal transduction. While Usp14 also deubiquitinates K63‐linked polyubiquitin chains on Dvl, its impact on Wnt signaling differs from that of CYLD [52]. Usp14 functions as a positive regulator of the Wnt signaling pathway. Notably, K63‐linked ubiquitination of Dvl occurs upon Wnt treatment and does not result in proteasomal degradation.

Praja ring finger ubiquitin ligase 2 (PJA2) targets TCF/LEF transcription factors for ubiquitination, leading to their downregulation [53]. PJA2 plays a crucial role in regulating stem cell differentiation by controlling the levels of TCF/LEF1. Nuclear prelamin A recognition factor (NARF) also induces the ubiquitination of TCF/LEF both *in vitro* and *in vivo*, functioning in conjunction with the E2 conjugating enzyme ubiquitin‐conjugating enzyme E2K (UBE2K) [54]. The ubiquitinated TCF/LEF proteins are subsequently degraded by the proteasome.

Several DUBs have been implicated in regulating Wnt signaling, including USP4, USP9X, USP47, and OTU deubiquitinase with linear linkage specificity (OTULIN). These enzymes positively influence β‐catenin stability, leading to enhanced β‐catenin‐mediated transcription and contributing to tumor cell growth or drug resistance. Given their roles in these processes, these DUBs represent potential therapeutic targets for cancer treatment [55, 56, 57, 58].

### 2.5 Core stem cell regulators: OCT4, SOX2, and NANOG

Cancer stem cells (CSCs), a subset of tumor cells with pluripotent tumorigenic potential, metastatic dissemination, drug resistance, and cancer recurrence, are regulated by a core set of TFs that govern stemness‐specific gene expression profiles, including the key stem cell regulators octamer‐binding transcription factor 4 (OCT4), SRY‐Box transcription factor 2 (SOX2), and NANOG [59]. These regulators are also subject to ubiquitination, a critical post‐translational modification that influences their stability and function. NANOG, a key TF for maintaining stem cell pluripotency and promoting somatic cell reprogramming, contains a proline (P), glutamic acid (E), serine (S) and threonine (T) (PEST) sequence that targets it for rapid degradation. ERK1 phosphorylation of S52 in NANOG facilitates its interaction with F‐box and WD repeat domain‐containing (FBXW)8, promoting its proteasomal degradation and contributing to stem cell differentiation [60]. Furthermore, phosphorylation of Nanog at S68 facilitates its recognition and polyubiquitination by speckle‐type POZ protein (SPOP), leading to its subsequent degradation via the proteasome [61, 62]. Mutations in SPOP that occur in cancer disrupt this regulatory mechanism, leading to the accumulation of NANOG and the promotion of cancer stem cell properties, ultimately contributing to prostate cancer progression [61, 62].

The protein levels of OCT4, a critical TF in stem cells, are tightly regulated. The WW domain containing E3 ubiquitin protein ligase 2 (WWP2) directly interacts with Oct4 and promotes its proteasomal degradation through ubiquitination [63]. In mouse embryonic fibroblasts (MEFs), WWP2‐mediated OCT4 ubiquitination represents a significant barrier to pluripotency induction. Disrupting WWP2‐mediated OCT4 ubiquitination during somatic cell reprogramming enhances OCT4 protein stability and leads to efficient induced pluripotent stem cell (iPSC) derivation [63]. Elevated OCT4 protein expression levels have been observed in various cancers, including cervical, prostate, colon, lung, and breast cancers [64]. Carboxy terminus of HSP70‐interacting protein (CHIP), an E3 ubiquitin ligase, was significantly downregulated in breast cancer stem cells. CHIP directly interacted with OCT4, and its overexpression led to decreased OCT4 stability through proteasomal degradation [65]. Indeed, patients with breast cancer exhibiting low CHIP expression demonstrated poor survival outcomes.

SOX2 is a pivotal TF involved in multiple stages of embryonic development and the maintenance of undifferentiated embryonic stem cells. Additionally, SOX2 is subject to regulation by the UPS in stem cells [66]. SET7/9 mono‐methylates SOX2 at K119, suppressing SOX2 transcriptional activity and promoting its ubiquitination and degradation [66]. WWP2 specifically recognizes K119‐methylated SOX2 through its HECT domain, facilitating its ubiquitination. Conversely, AKT1 phosphorylation of SOX2 at threonine 118 (T118) stabilizes SOX2 by counteracting SET7/9‐mediated methylation. This dynamic interplay between methylation and phosphorylation regulates SOX2 stability in embryonic stem cells (ESCs) and plays a crucial role in lineage specification during differentiation [66]. The cullin‐4A (CUL4A)^DET1‐COP1^ complex and the deubiquitinase OTUD7B also govern SOX2 protein stability during neural progenitor cell (NPC) differentiation. SOX2 expression levels inversely correlate with CUL4A and COP1 levels, while exhibiting a reciprocal relationship with OTUD7B expression during NPC differentiation [67]. SOX2 amplification and aberrantly increased expression have been observed in various human cancers [68]. Specifically, SOX2 is crucial for tumor initiation and CSC function in squamous cell carcinomas, including esophageal squamous cell carcinoma. Interestingly, SOX2 is also regulated by CHIP, an E3 ubiquitin ligase primarily known for its role in regulating OCT4. CHIP interacts with SOX2 primarily through the chaperone HSP70, facilitating SOX2 ubiquitination and subsequent degradation via the proteasome [69]. Conversely, HSP90 promotes SOX2 stability, and inhibiting HSP90 activity can induce SOX2 ubiquitination and degradation [69].

### 2.6 NF‐κB

Nuclear factor kappa B (NF‐κB) plays a pivotal role in diverse cellular processes, including cell cycle regulation, immune response, and malignant transformation [70]. The UPS serves as a primary regulator of NF‐κB activation, which is initiated by the phosphorylation‐induced ubiquitination and degradation of inhibitory κB (IκB) proteins [71]. This allows NF‐κB dimers to translocate to the nucleus and exert regulatory effects on the transcription of numerous target genes. NF‐κB is constitutively present in the cytoplasm of resting cells in a latent form complexed with IκB proteins, facilitating its rapid activation upon stimulation by proinflammatory cytokines, oxidative stress, or other signaling cues. IκB proteins are phosphorylated by IκB kinase (IKK), leading to their ubiquitination by SCF^β‐TrCP^ and degradation by the proteasome, releasing NF‐κB dimers for nuclear translocation [72, 73]. Multiple studies have demonstrated upregulated NF‐κB activity in various malignancies [74]. For example, enhanced expression of NF‐κB p65 (RelA) in prostate adenocarcinoma has been correlated with increased tumor grade and aggressiveness [75]. Additionally, ovarian carcinoma and borderline ovarian tumors exhibit a higher expression of NF‐κB p50 and p65 compared to benign ovarian tumors [76].

As NF‐κB primarily functions as a heterodimer composed of p65 and p50 subunits, this review focuses on the regulation of p65 protein stability. FBXW2 directly binds to p65, promoting its ubiquitination and degradation [77]. This process suppresses breast cancer stemness, tumorigenesis, and resistance to paclitaxel. Interestingly, histone acetyltransferase p300‐mediated acetylation of p65 inhibits FBXW2‐induced ubiquitination, suggesting a regulatory interplay between these two modifications. In lung adenocarcinoma, RNF182, another E3 ubiquitin ligase, also promotes p65 ubiquitination and degradation [78]. Given that NF‐κB p65 is a transcriptional activator of programmed cell death ligand 1 (PD‐L1), overexpression of RNF182 can lead to a decline in PD‐L1 expression through p65 degradation. TRIM7 is frequently downregulated in various cancers compared to normal tissues, suggesting a potential role as a tumor suppressor protein [79]. TRIM7 directly interacts with NF‐κB p65 in lung cancer, promoting its ubiquitination and subsequent degradation [80]. The stability of NF‐κB p65 is influenced by its methylation status [81]. LSD1, a histone demethylase, can interact with p65 in its phosphorylated form, leading to demethylation of specific lysine residues, K314/315, and subsequent enhancement of p65 protein stability [82].

**Fig. 2:**
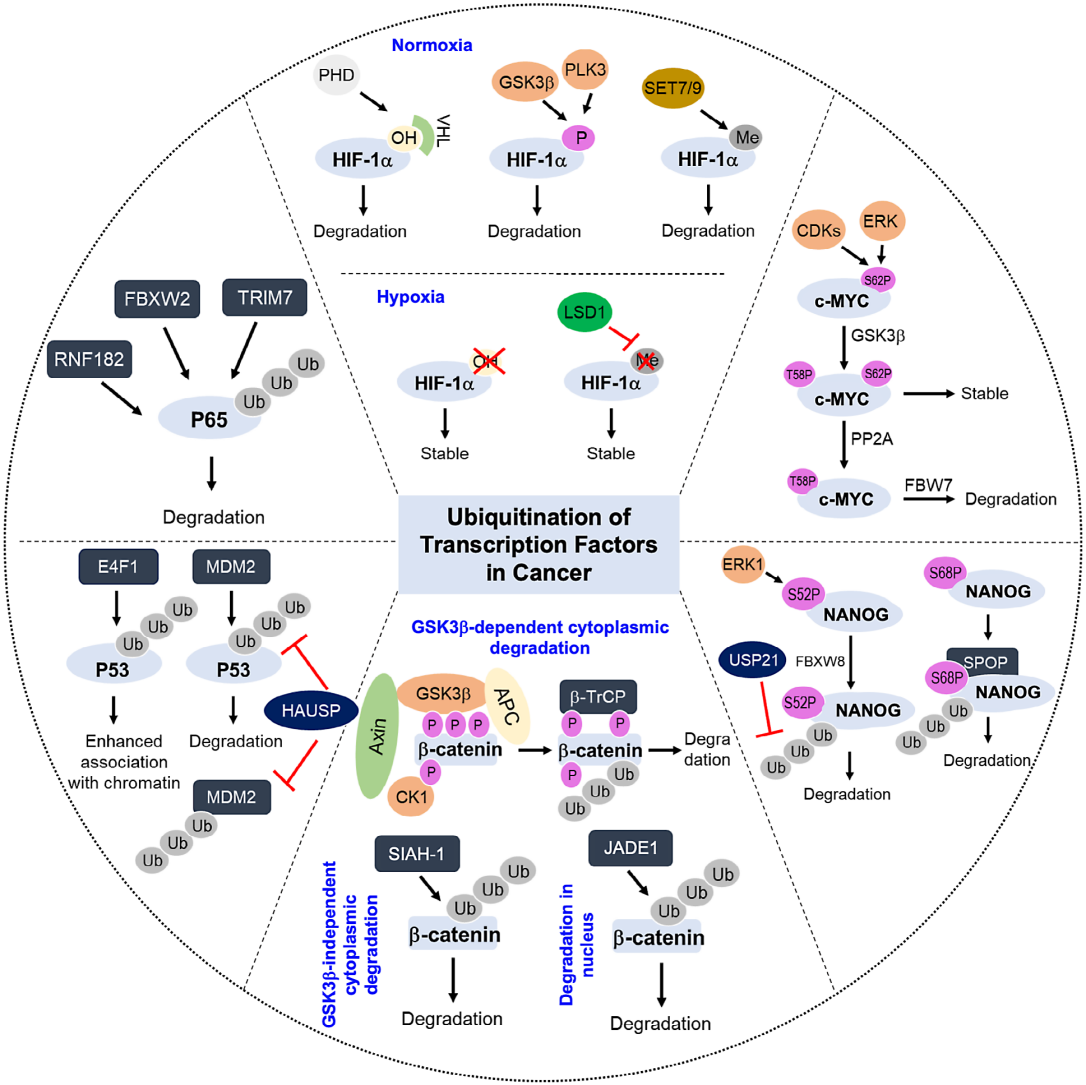
Ubiquitination of transcription factors in cancer. The UPS, in conjunction with post‐translational modifications such as phosphorylation, hydroxylation, and methylation, precisely regulates the stability and subcellular localization of transcription factors.

Each TF that plays a crucial role in different types of cancer is influenced by ubiquitination‐dependent stabilization through distinct mechanisms (Fig. 2). Consequently, targeting the mechanisms of various E3s and DUBs involved in this process offers promising therapeutic potential for cancer treatment.

## 3 UPS‐targeting anticancer drugs

**Fig. 3:**
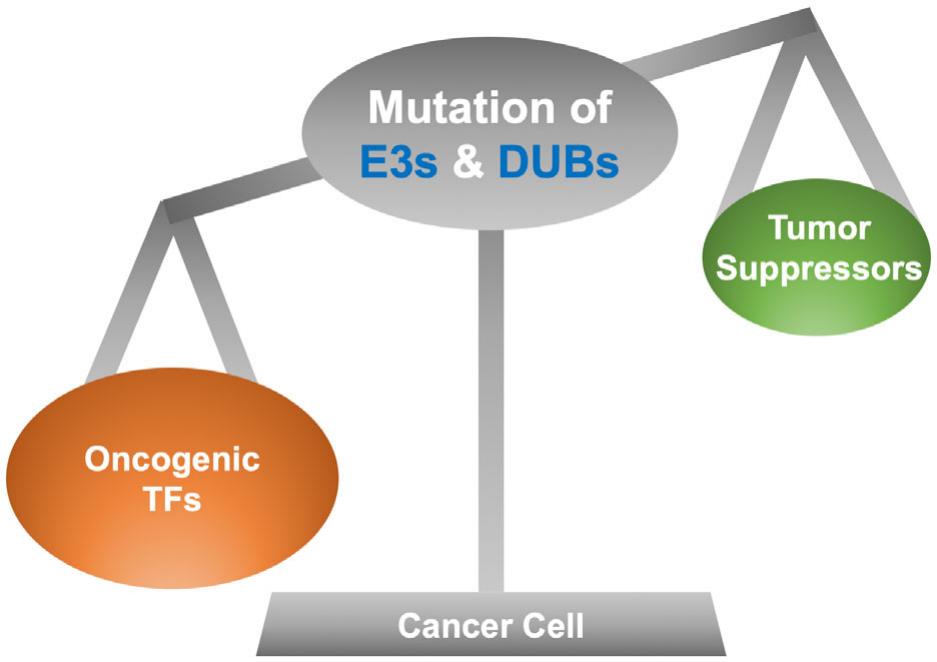
Imbalance of E3s and DUBs in cancer cells. In cancer cells, mutations in E3s and DUBs disrupt the balance in the stability of oncogenic transcription factors and tumor suppressors, promoting cancer progression.

The UPS plays a crucial role in maintaining cellular protein homeostasis, making it an attractive target for anticancer therapy [83, 84, 85]. Among UPS‐targeting inhibitors, 26S proteasome inhibitors such as bortezomib and carfilzomib have shown significant efficacy in treating hematologic malignancies by blocking protein degradation and inducing apoptosis (Table 1). Additionally, inhibitors targeting E3s or DUBs offer an alternative strategy to modulate specific protein turnover, providing a more selective approach to cancer treatment (Fig. 3). These emerging inhibitors hold great potential for overcoming drug resistance and improving therapeutic outcomes in various cancers.

**Table 1:** 26S proteasome inhibitors

Structural class	Proteasome inhibitor	Structure	Binding mode	Treatment	Clinical trials/approval
Peptide boronic acid	Bortezomib (Velcade)	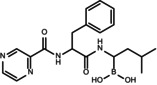	Reversible	Multiple myeloma, mantle cell lymphoma	FDA approved in 2003
	Delanzomib (CEP‐18770)	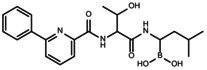	Reversible	Multiple myeloma, solid tumors or non‐Hodgkin's lymphomas	Phase I/II (NCT01348919, NCT00572637, NCT01023880)
	Ixazomib (Ninlaro)	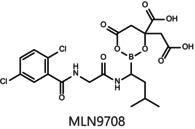 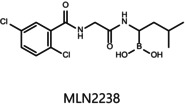	Reversible	Relapsed/refractory multiple myeloma	FDA approved in 2015
Peptideepoxyketone	Carfilzomib (Kyprolis)	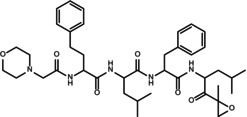	Irreversible	Relapsed/refractory multiple myeloma	FDA approved in 2012

Since the success of proteasome inhibitors such as bortezomib in treating multiple myeloma, there has been a surge of interest in targeting the UPS for cancer therapy. A new generation of therapeutic strategies focuses on targeting specific ubiquitin pathway enzymes, such as E1, E2, and E3 enzymes, rather than broadly inhibiting the proteasome. This approach offers the potential for greater specificity and reduced side effects. Given their substrate specificity, E3 ligases have emerged as particularly attractive targets for cancer therapy, leading to increased efforts to develop small molecules that selectively inhibit these enzymes.

### 3.1 Targeting p53‐MDM2 interaction by inhibitors

MDM2, an E3 ubiquitin ligase containing a RING domain, plays a critical role in regulating p53 stability and function. One promising strategy to restore p53 function is to disrupt the p53‐MDM2 interaction using small molecules [86]. Currently, nine small molecules targeting the p53‐MDM2 interface are undergoing clinical evaluation (Table 2). Numerous global pharmaceutical companies are actively engaged in the development of novel drugs with exceptional efficacy. Among the drugs currently undergoing clinical trials, RG7388 progressed to Phase 3 (NTC 02545283) but was ultimately discontinued due to a lack of efficacy, as determined by interim analysis. APG‐1115 and HDM201 are currently being evaluated in Phase 2 clinical trials.

**Table 2:** p53‐MDM2 interaction inhibitors

Drug	Structure	Treatment	Most advanced clinical trial	Sponsor
RG7112 (RO5045337)	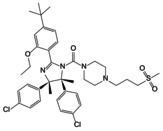	Advanced solid tumors, hematologic neoplasm, solid tumors, sarcoma, AML, CML	Phase I (NCT00559533, NCT00623870)	Roche
RG7388 (Idasanutlin)	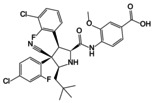	Solid tumors, relapsed and refractory AML, relapsed and refractory follicular lymphoma, relapsed and refractory diffuse large B‐cell lymphoma, AML, breast cancer, solid tumors, neoplasms, relapsed multiple myeloma, solid tumors, colorectal cancer, glioblastoma, non‐Hodgkin's lymphoma	Phase III (NCT02545283: the study was stopped for futility based on efficacy results at the interim analysis)	Roche
AMG232 (KRT‐232)	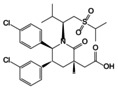	Advanced solid tumors, multiple myeloma, AML, relapsed and refractory AML, metastatic melanoma, soft tissue sarcoma, polycythemia vera, brain cancer	Phase Ib/IIa (NCT02110355)	Kartos Therapeutics
APG‐115 (AA‐115)	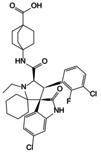	Advanced solid tumors, lymphomas, metastatic melanomas, salivary gland carcinoma, AML, acute lymphocytic leukemia, neuroblastoma, liposarcoma, T‐prolymphocytic leukemia	Phase II (NCT03781986, NCT04785196, NCT04358393, NCT04496349, NCT03611868)	Univ. of Michigan, Rogel Cancer Center, Ascentage Pharma Group Inc.
CGM‐097	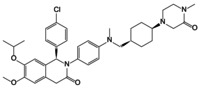	Advanced solid tumors with TP53wt	Phase I (NCT01760525)	Novartis
HDM201	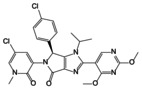	Liposarcoma, uveal melanoma, advanced solid and hematological TP53wt tumors, AML, advanced/metastatic colorectal cancer, myelofibrosis, colorectal cancer, nonsmall cell lung carcinoma, triple‐negative breast cancer, renal cell carcinoma, malignant solid tumors, myelodysplastic syndromes	Phase II (NCT04116541, NCT05180695)	Novartis
DS‐3032b (Milademetan)	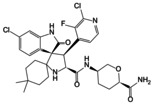	Advanced solid tumors, lymphomas, relapsed and refractory AML, AML, myelodysplastic syndromes, myeloma	Phase II (NCT03634228: this study was terminated early due to lack of adequate response)	M.D. Anderson Cancer Center
SAR405838	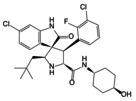	Neoplasm malignant	Phase I (NCT01636479, NCT01985191)	Sanofi S.A.

### 3.2 Targeting enzymatic activity of E3s or DUBs

E3 ubiquitin ligases, as the terminal enzymes in the ubiquitination cascade, confer substrate specificity. To date, ~600 E3 ligases have been identified, each capable of recognizing and ubiquitinating a subset of substrates. Consequently, targeting a particular E3 ligase is expected to selectively affect the pathways regulated by that enzyme. While RING domain‐containing E3 ligases lack intrinsic catalytic activity, they function as scaffolds, facilitating the interaction between E2 enzymes, ubiquitin, and substrates [87]. Therefore, targeting RING E3 ligases often necessitates the development of allosteric or protein–protein interaction inhibitors, similar to those used to disrupt the MDM2‐p53 interactions [88]. In contrast, HECT E3 ligases possess intrinsic enzymatic activity, making their inhibition more straightforward by targeting the catalytic site. Despite extensive research efforts, the identification of E3 inhibitors has been limited, partly due to the historical focus on disrupting enzyme–substrate interactions, which are considered more challenging to target than catalytic sites. One potential concern when targeting the enzymatic activity of E3 ligases is their propensity for auto‐ubiquitination [89]. Consequently, inhibiting the catalytic activity of E3 ligases may inadvertently stabilize both the E3 protein itself and its substrates. Recent advancements in drug discovery have led to the identification of small‐molecule inhibitors targeting Cullin‐RING E3 ubiquitin ligases (CRLs). Through high‐throughput screening and medicinal chemistry optimization, two compounds, 33–11 and KH‐4‐43, have been developed that specifically inhibit CRL4 [90]. These compounds bind to the core catalytic complex of CRL4, disrupting its ubiquitination activity and resulting in the stabilization of its substrate, chromatin licensing and DNA replication factor 1 (CDT1). Furthermore, treatment with 33‐11 or KH‐4‐43 demonstrated cytotoxic effects in a panel of 36 tumor cell lines [91]. While not specifically designed as an anticancer drug, a recent publication describes an inhibitor of SMAD‐specific E3 ubiquitin protein ligase 1 (SMURF1). The potent small‐molecule SMURF1 ligase inhibitor, 38, has exhibited favorable oral pharmacokinetics in rats and significant efficacy in a rodent model of pulmonary arterial hypertension [91].

DUBs, often overexpressed in various cancers, regulate critical substrates involved in cancer‐associated processes, making them promising targets for alternative cancer therapies [92]. USP14, a DUB implicated in promoting tumorigenesis in multiple cancer types, interacts with and stabilizes AR proteins [93]. Inhibiting USP14 in prostate cancer cells can lead to AR‐dependent cell cycle arrest, suggesting its potential therapeutic value [94]. Moreover, overexpression of USP14 in multiple myeloma cells significantly enhances cell adhesion‐mediated drug resistance through the upregulation of Wnt signaling [95]. The small‐molecule VLX1570 specifically inhibits the activity of USP14 and ubiquitin C‐terminal hydrolase L5 (UCHL5) within the 19S regulatory subunit of the proteasome. This results in the rapid accumulation of high molecular weight ubiquitin conjugates, proteasome shutdown, and robust antitumor activity in various preclinical models of cancer, including multiple myeloma, lymphoma, Ewing's sarcoma, and others [96, 97]. VLX1570 was the first DUB inhibitor to enter clinical trials, demonstrating enhanced potency and improved aqueous solubility. However, a Phase 1 clinical trial (NCT02372240) in relapsed and/or refractory multiple myeloma patients was discontinued due to toxicity issues [98]. Continued efforts to identify DUB inhibitors with greater therapeutic potential are warranted, given the promising preclinical antitumor activity observed with compounds like VLX1570. KSQ‐4279 (RO7623066), a USP1 inhibitor, is currently undergoing Phase I clinical trials for the treatment of patients with advanced solid tumors (NCT05240898). In preclinical models, KSQ‐4279 has shown therapeutic potential in combination with olaparib for treating patients who are either intrinsically resistant or have developed acquired resistance to poly(ADP‐ribose) polymerase (PARP) inhibitors [99].

Most of the current clinically approved drugs for cancer treatment are proteasome inhibitors targeting the UPS. However, these inhibitors have a limitation, in that they cannot selectively target specific transcription factors. Therefore, novel strategies are required to precisely target specific transcription factors. ‘RG7388 (NTC 02545283)’, is a drug that inhibits the binding of p53 and MDM2 through PPI inhibitors rather than directly regulating E3s or DUBs. Thus, there is a need to develop new therapeutic strategies that can directly regulate the ubiquitination and degradation of specific transcription factors.

## 4 Targeted degradation of transcription factors by PROTAC

**Fig. 4:**
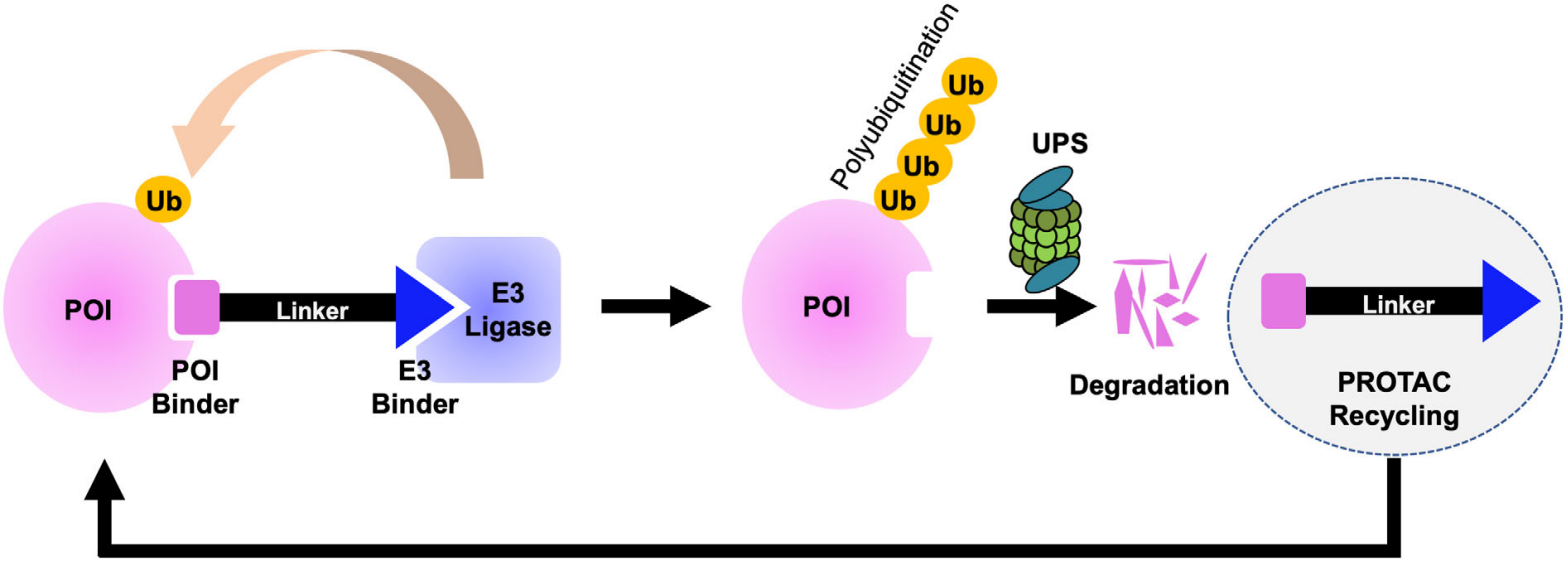
Schematic model of a PROTAC that links the E3 ligase to the protein of interest (POI). PROTACs are heterobifunctional small molecules composed of two distinct ligands connected by a linker. One moiety binds to a protein of interest (POI), while the other binds to an E3 ubiquitin ligase. This dual binding facilitates the recruitment of the POI to the E3 ligase, leading to its polyubiquitination and subsequent degradation by the proteasome. Following the degradation of the POI, the PROTAC molecule can be recycled for subsequent rounds of target protein degradation.

PROteolysis TArgeting Chimeras (PROTACs) have emerged as a promising approach for selectively degrading specific disease‐associated proteins by leveraging the cell's own protein degradation machinery [100]. A PROTAC molecule consists of an E3 ligase‐recruiting ligand, a protein of interest (POI)‐targeting warhead, and a flexible linker connecting the two [101] (Fig. 4). By simultaneously binding to the POI and an E3 ligase, the PROTAC induces the ubiquitination of the POI, leading to its subsequent degradation by the UPS. Importantly, the PROTAC molecule is recycled after facilitating the degradation of the POI, allowing it to target additional copies of the protein [102]. Since the concept of targeted protein degradation (TPD) was first introduced in 1999 [103], significant advancements have been made, and several PROTAC molecules are currently undergoing clinical evaluation.

As a novel therapeutic modality, PROTACs represent a heterobifunctional degrader class that requires rigorous evaluation in clinical trials to establish proof of concept [100]. Key areas of interest include drug‐like properties, safety, target protein degradation, and therapeutic efficacy.

### 4.1 Targeted degradation of nuclear receptors associated with cancers

The androgen receptor (AR) and estrogen receptor (ER) are frequently overexpressed or mutated in prostate and breast cancers, respectively. Due to their involvement in key pro‐oncogenic processes, such as cellular proliferation and survival, they serve as promising therapeutic targets in cancer treatment. These nuclear receptors contain ligand‐binding domains (LBDs), which facilitate the development of antagonists or selective modulators capable of effectively inhibiting their activation. Selective AR and ER antagonists bind to the LBD, preventing the conformational changes required for coactivator recruitment. Instead, this conformational alteration promotes the recruitment of corepressors, thereby suppressing the expression of target genes [104]. Building upon the discovery of AR and ER modulators, PROTAC‐based AR and ER degraders have been developed to enhance therapeutic efficacy. AR and ER degraders facilitate the targeted degradation of both wildtype and mutant receptor forms, offering a promising strategy to overcome the drug resistance commonly associated with conventional small‐molecule inhibitors [105]. In 2020, initial positive data from Phase I trials of ARV‐110 and ARV‐471 provided affirmative answers to these foundational questions. ARV‐110, an AR degrader, and ARV‐471, an ER degrader, demonstrated favorable safety profiles, efficacious exposure, and signs of clinical efficacy, solidifying the therapeutic potential of the PROTAC modality [106].

Additionally, a STAT3 degrader was developed through the optimization of a STAT3 SH2 domain inhibitor. As STAT3 is a critical transcription factor involved in tumor development and progression, it has emerged as a promising therapeutic target in cancer treatment. Treatment with the STAT3 degrader effectively induces STAT3 protein degradation both *in vitro* and *in vivo*, leading to robust suppression of its transcriptional network in leukemia and lymphoma cells and resulting in sustained tumor regression [107]. The STAT3 degrader, KT‐333, is currently undergoing Phase I clinical trials for the treatment of peripheral T‐cell lymphoma (PTCL), cutaneous T‐cell lymphoma (CTCL), large granular lymphocytic leukemia (LGL‐L), and solid tumors (NCT05225584).

Notably, the majority of PROTACs in clinical development utilize cereblon (CRBN) or VHL as the E3 ligase, highlighting its emergence as a preferred choice for the initial wave of TPD therapeutics [108] (Table 3). However, with over 600 human ubiquitin E3 ligases identified, the development of new E3 ligase‐based PROTACs could expand the range of targetable proteins and potentially enhance therapeutic efficacy.

**Table 3:** PROTAC drugs under clinical trials. The PROTAC molecule consists of three main components: a protein of interest (POI) binder (pink), an E3 ligase binder (blue), and a flexible linker (black) connecting the two. AR, androgen receptor; BTK: Bruton's tyrosine kinase; CRBN, cereblon; ER, estrogen receptor.

Drug	Structure	E3 binder	Target POI	Treatment	Most advanced clinical trial	Sponsor
ARV‐110 (Bavdegalutamide)	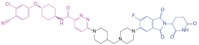	CRBN	AR	Metastatic castration resistant prostate cancer	Phase II (NCT03888612)	Arvinas
ARV‐471 (PF‐07850327, Vepdegestrant)	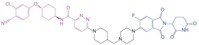	CRBN	ER	ER(+)/HER2(−) advanced breast cancer	Phase III (NCT05909397, NCT05654623)	Pfizer
NX‐2127	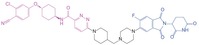	CRBN	BTK, IKZF1 (Ikaros), IKZF3 (Aiolos)	Relapsed/refractory B‐cell malignancie	Phase I (NCT04830137)	Nurix Therapeutics
KT‐474 (SAR444656)	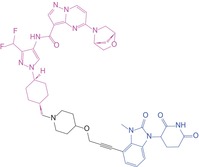	CRBN	IRAK4	Atopic dermatitis or hidradenitis suppurativa	Phase I (NCT04772885)	Kymera Therapeutics
DT2216	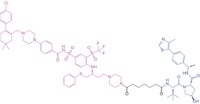	VHL	BCLXL	Relapsed/refractory malignancies	Phase I (NCT04886622)	Dialectic Therapeutics
CC‐94676	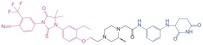	CRBN	AR	Metastatic castration resistant prostate cancer	Phase I (NCT04428788)	Celgene
NX‐2127	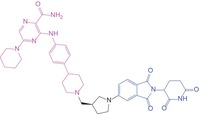	CRBN	BTK	Relapsed/refractory B‐cell malignancies	Phase I (NCT04830137)	Nurix Therapeutics
NX‐5948	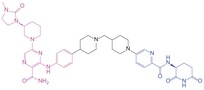	CRBN	BTK	Relapsed/refractory B‐cell malignancies	Phase I (NCT05131022)	Nurix Therapeutics
FHD‐609	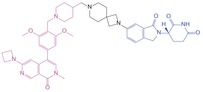	CRBN	BRD7/9	Advanced synovial sarcoma or advanced SMARCB1‐loss tumors	Phase I (NCT04965753)	Foghorn Therapeutics

### 4.2 Targeted degradation of transcription factors by oligonucleotide‐based PROTACs

Many transcription factors have traditionally been considered undruggable due to the structural characteristics of their protein–protein interaction surfaces, which are typically flat and lack the deep binding pockets found in enzyme active sites [104]. This structural limitation has made the development of small‐molecule inhibitors particularly challenging, except for specific nuclear receptors and a few transcription factors. Recently, an innovative strategy has emerged that utilizes the DNA binding motif of transcription factors as a warhead in PROTAC‐based approaches, offering a novel avenue for targeted protein degradation (Table 4).

**Table 4:** Targeted degradation of transcription factors by oligonucleotide‐based PROTAC. The oligonucleotide PROTAC molecule consists of three main components: DNA sequence, an E3 ligase binder, and a flexible linker connecting the two. Bold DNA sequences are DNA binding motifs. CRBN, cereblon. *phosphorothioation, o: internal C3 spacer.

Target	DNA sequences	Name	E3	Tested cell/model	References
NF‐κB/p65	Single strand DNA oligonucleotide with hairpin structure 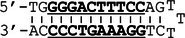	dNF‐κB	VHL	HeLa cell (cervical cancer cell)	[110]
E2F1	Annealed oligo to form a double stand DNA 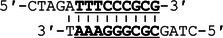	dE2F	VHL	HeLa cell (cervical cancer cell)	[110]
LEF1	Annealed oligo to form a double stand DNA with three extra nucleotides for protection of oligo degradation 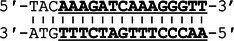	LEF1 OP‐V1	VHL	PC3, DU145 (prostate cancer cell)	[111]
ERG	Annealed oligo to form a double stand DNA with three extra nucleotides for protection of oligo degradation 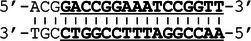	ERG OP‐C‐N1	CRBN	VCaP (prostate cancer cell)	[111]
STAT3	CpG sequence (TLR9 ligands)‐conjugated, single strand DNA oligonucleotide with hairpin structure 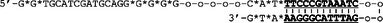	C‐STAT3D^PROTAC^	CRBN	DLBCL cells (non‐Hodgkin lymphoma cell), OCI‐Ly18 (B cell lymphoma cell), xenograft model	[112]
STAT3	Annealed oligo to form a double stand DNA 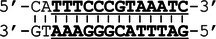	POM‐STAT3	CRBN	NCI‐H2087 (lung cancer cell line), MCF7 (breast cancer cell)	[114]
ER	Single strand DNA oligonucleotide with hairpin structure 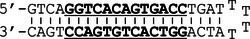	LCL‐ER(dec)	IAP	MCF7 (breast cancer cell)	[113]

By leveraging the intrinsic property of transcription factors to bind directly to consensus DNA sequences, the challenge of developing small‐molecule inhibitors—due to the lack of ligand‐binding pockets—has been effectively addressed. This innovative approach, which bridges oligonucleotides with E3 ligase‐binding moieties, has been explored in several studies [105].

As a proof of concept, HaloPROTAC‐mediated degradation of transcription factors such as p50 and brachyury was successfully demonstrated [109]. Shortly thereafter, the development of VHL‐based TF‐PROTACs, including NF‐κB‐PROTAC (dNF‐κB) and E2F‐PROTAC (dE2F), was reported [110]. These TF‐PROTACs effectively induced the degradation of endogenous p65 and E2F1 proteins in cells, leading to potent antiproliferative effects. Furthermore, oligonucleotide‐based PROTACs have been successfully utilized to degrade other cancer‐associated transcription factors, such as lymphoid enhancer‐binding factor 1 (LEF1) and ETS‐related gene (ERG) [111]. The degradation of these proteins impaired their transcriptional activity and inhibited cancer cell growth both *in vitro* and *in vivo*. Similarly, STAT3 has also been targeted using oligonucleotide‐based PROTAC strategies [112].

As research in this field advances, strategies to enhance efficacy and cell selectivity have been actively explored. Recent studies have investigated the impact of nucleotide modifications and oligonucleotide duplex structures on PROTAC function. Phosphorothioate (PS) modification of nucleotides improved nuclease resistance, but reduced target specificity, leading to nonspecific degradation [113]. In contrast, the incorporation of a T4 loop hairpin structure demonstrated sustained ERα degradation activity. Rational design of nucleic acid properties could further optimize the potency and specificity of oligonucleotide‐based PROTACs [113]. Moreover, conjugation of Toll‐like receptor 9 (TLR9) ligands, such as CpG oligonucleotides, with the STAT3‐binding motif enabled selective targeting of TLR9+ immune and cancer cells [113]. When combined with a TLR9‐directed delivery strategy, this approach enhanced STAT3‐selective degradation, resulting in improved antitumor efficacy and safety.

Oligonucleotide‐based PROTACs offer a versatile and generalizable strategy for targeting traditionally undruggable transcription factors by leveraging DNA sequences. Since oligonucleotides are already employed in therapies such as antisense oligonucleotides (ASOs), existing strategies to enhance stability, tissue penetration, and reduce immunogenicity can be applied to this platform [114]. However, challenges remain, particularly in delivery, due to their strong negative charge, as well as suboptimal pharmacokinetics and relatively low potency, which may hinder clinical translation. Therefore, further research aimed at overcoming these limitations will be essential for their future development.

## 5 Conclusion

UPS plays a critical role in regulating protein homeostasis and is frequently dysregulated in cancer. Transcription factors, including p53, c‐MYC, HIF‐1α, and NF‐κB, are key targets of ubiquitination, and their aberrant regulation contributes to tumorigenesis. While proteasome inhibitors have been approved for clinical use, the development of more specific inhibitors targeting E3s remains a significant challenge due to their complex and redundant nature.

To address these challenges, promising strategies include the development of PPI inhibitors and PROTAC technology. PPI inhibitors targeting specific TF‐E3 ligase interactions, such as the p53‐MDM2 interface, offer a high degree of specificity and have shown promising results in preclinical and clinical studies. PROTACs, which utilize E3s to degrade target proteins, also represent a promising approach for cancer therapy. Furthermore, exploring the combination of E3 inhibitors with other therapeutic modalities, such as chemotherapy or immunotherapy, may enhance therapeutic efficacy and overcome resistance. Advances in our understanding of the UPS and its role in cancer will ultimately lead to the development of more effective and targeted therapies for patients.

Since E3s are involved in numerous cellular processes, inhibiting them can result in off‐target effects, potentially disrupting normal cellular functions and leading to toxicity. Moreover, our incomplete knowledge of the full range of substrates regulated by specific E3s limits our ability to accurately predict and control the outcomes of E3 inhibition in cancer therapy. Continuous efforts are therefore needed to identify new TFs‐E3s combinations that could serve as a foundation for developing cancer therapies. To overcome challenges such as off‐target effects when directly targeting E3s, there should be an increased focus on utilizing bioinformatics tools and large‐scale databases. This approach will help clarify the mechanisms between E3s and TFs, ensuring greater target specificity. In turn, this will be essential for developing inhibitors that precisely target transcription factors involved in cancer.

## Conflict of interest

The authors declare no conflict of interest.

## Author contributions

DK: conceptualization, writing – original draft, reviewing & editing, visualization, funding acquisition. HJN: conceptualization, writing – original draft, reviewing & editing, visualization, funding acquisition. SHB: conceptualization, reviewing & editing, supervision, funding acquisition.
